# Short-term safety and Long-term efficacy of multivisceral resection in pT4b gastric cancer patients without distant metastasis: a 20-year experience in China National Cancer Center

**DOI:** 10.7150/jca.75456

**Published:** 2022-08-15

**Authors:** Xiaojie Zhang, Wanqing Wang, Lulu Zhao, Penghui Niu, Chunguang Guo, Dongbing Zhao, Yingtai Chen

**Affiliations:** Department of Pancreatic and Gastric Surgical Oncology, National Cancer Center/National Clinical Research for Cancer/Cancer Hospital, Chinese Academy of Medical Sciences and Peking Union Medical College, Beijing 100021, China.

**Keywords:** gastric cancer, T4b, multivisceral resection (MVR), postoperative complications, recurrence, survival

## Abstract

**Background:** Multivisceral resection is occasionally necessary for pT4b gastric cancer patients to achieve negative margin. The purpose of this study is to assess the short-term safety and long-term efficacy of this approach.

**Methods:** A single-center, retrospective analysis was conducted for pT4b gastric cancer patients after curative-intent multivisceral resection from the China National Cancer Center Gastric Cancer Database (NCCGCDB) from 1998 to 2018. The postoperative complications, recurrence patterns, long-term survival, and prognostic factors were analyzed.

**Results:** A total of 210 patients were included in the study. The most common combined resection organs were multiple organs (30.5%), pancreas (20.5%), colon (16.7%), and liver (9.0%). Seventeen patients (8.1%) developed postoperative complications and hospital death was observed in one patient (0.5%). The most common postoperative complications were anastomotic leak (4.3%) and intra-abdominal infection (5.7%). The 3-year and 5-year disease-free survival (DFS) rates for the patients investigated were 38.0% and 33.8%, respectively, and the 3-year and 5-year overall survival (OS) rates were 48.2% and 39.1%, respectively. Multivariate Cox regression analysis proved that negative nerve invasion was independent risk factors for DFS (HR: 2.202, 95%CI: 1.144-4.236, *P*=0.018) and OS (HR: 2.219, 95%CI: 1.164-4.231, *P*=0.015).

**Conclusions:** Multivisceral resection in pT4b gastric cancer patients without distant metastasis was effective and had an acceptable safety profile.

## Background

Gastric cancer (GC) is one of the most common fatal malignancies with high risk of metastasis and tumor recurrence 1. Considering that locally advanced GC sometimes invade the surrounding organs (T4b), such as pancreas, colon, and liver 2, the multivisceral resection (MVR) surgery is necessary to achieve a negative margin 3, 4. Generally, MVR surgery is considered to have higher cost with increased risk of postoperative complications and mortality 5. The short-term safety and long-term efficacy have been widely debated over the years.

Notably, most of the previous studies included the patients with clinical T4b (cT4b) 2, 4, 6-10. However, in some cT4b patients, the tumor itself did not directly invade the surrounding organs due to the inflammatory response 6. Therefore, partial GC patients with cT4b included in the previous study (pathologically confirmed T4a, pT4a) might not require extended MVR. Moreover, the previous study demonstrated that the median overall survival of GC patients with pT4a were higher than pT4b who underwent MVR surgery (22.6 months vs. 17.7 months) 6.

To date, only a few studies evaluated the safety and efficacy of MVR surgery focusing on pathologic T4b (pT4b) patients 3, 6, 11. In Korea Cancer Center Hospital, 243 GC patients with pT4b were retrospectively reviewed, and the results demonstrated that the postoperative mortality rate was 0.8% and the media overall survival (OS) was 26 months 11. Another National Cancer Database (NCDB) study showed that the mortality rate within 30 days of MVR surgery was 7.5% and the media OS was 12.9 months in pT4b GC patients underwent MVR surgery 6. However, neither of the two studies explored the recurrence pattern of pT4b GC patients after MVR surgery. Meanwhile, the rates of postoperative complications and mortality varies greatly.

Therefore, we designed this study to assess the short-term safety, recurrence pattern and long-term survival of MVR surgery in pT4b GC patients based on the China National Cancer Center Gastric Cancer Database (NCCGCDB).

## Materials and Methods

### Patients

We retrospectively collected the clinicopathologic characteristics of pT4b patients who underwent potential curative MVR surgery from the China National Cancer Center Gastric Cancer Database (NCCGCDB). The details of NCCGCDB have been previously described and recognized 12. The inclusion criteria were as follows: (i). Age more than 18 years; (ii). Adenocarcinoma of stomach; (iii). Postoperative pathology confirmed T4b. The exclusion criteria included: (i). Patients with other tumor history; (ii). Patients who confirmed with distant metastasis; (iii). Patients with incomplete clinical data.

The definition of pT4b was that gastric cancer directly invaded the adjacent structures, including the spleen, colon, liver, diaphragm, pancreas, abdominal wall, adrenal gland, kidney, small intestine, and retroperitoneum, according to the guidelines of the National Comprehensive Cancer Network (NCCN, version 5.2021). The requirement for written informed consent by patients was waived due to the retrospective nature of the study. Eventually, a total of 210 patients were enrolled into the final analysis.

### Short-term and Long-term outcomes

The main short-term outcomes were operative difficulty, postoperative complications, and perioperative mortality. The operative difficulty was reflected from the aspects of operative time, blood transfusion, and postoperative hospital stay. The main long-term outcomes were disease-free survival time (DFS) and overall survival time (OS). DFS was defined as the time from surgery to the locoregional and systemic recurrence. OS was defined as the time from surgery to the death or last follow-up. Recurrence pattern was classified as locoregional recurrence, peritoneal metastasis, and distant metastasis, which has been described in detail in previous studies 13, 14.

### Postoperative follow-up

The postoperative follow-up was performed through outpatient clinical visits, telephone contact, and death registries. Finally, 53 patients were lost to follow-up and the follow-up rate was 74.8%. The median duration of follow-up was 22 months (rang, 1-192 months).

### Statistical analysis

The basic clinicopathologic features of the patients was presented using descriptive statistics. Categorical variables were presented with counts and proportions, while continuous variables were presented with medians and standard deviation (SD). Categorical variables were compared using the chi-square test and Fisher's exact test. Continuous variables were compared using the Mann-Whitney U test. Survival analysis was conducted using the Cox proportional hazards model. In the multivariate models, we included the factors with *P*≤0.2 in the univariate analysis and other important factors might affect the survival outcomes. All the survival analysis was performed with the SPSS software (SPSS Inc., Chicago, IL, USA, version 22.0). The survival curves were depicted according to the Kaplan-Meier method through GraphPad Prism software (GraphPad Software, La Jolla, CA, USA, version 8.0.2). Results with a two-tailed *P*<0.05 were considered as statistically significant.

## Results

### Clinicopathologic characteristics

The basic clinicopathologic characteristics of the 210 pT4b GC patients who underwent MVR surgery were displayed in the **Table 1**. The median age of all patients was 61 years (range, 24-82 years). Most patients (87.1%) were proved to have locoregional lymph nodes metastasis. In addition, most pT4b patients had large tumor size and poor differentiation. The most common combined resection organs were multiple organs (30.5%), pancreas (20.5%), colon (16.7%), and liver (9.0%). Although all the patients underwent potentially radical surgery, 12 patients (5.7%) were confirmed to have the positive surgical margins.

### Short-term outcomes

Regarding the surgical difficulty, as shown in the **Table 1**, most patients (93.3%) received open operation, and only 14 patients (6.7%) received laparoscopic surgery. The mean operation time were 209.8 minutes in all patients, and the operation time was longer in patients underwent gastrectomy combined with spleen resection. More than 50% of the patients required intraoperative blood transfusion, especially for patients combined pancreas resection and combined liver resection. Moreover, patients who received combined pancreas resection and multiple organs resection were more likely to have a longer stay in hospital (**Table 2**).

In terms of surgical safety, 17 patients (8.1%) developed postoperative complications and hospital death was observed in 1 patient (0.5%) due to intraabdominal bleeding. The most common postoperative complications were anastomotic leak (4.3%) and intra-abdominal infection (5.7%). Combined resection of pancreas and multiple organs have higher risk of postoperative complications and mortality.

### Long-term survival outcomes

**Figure 1:**
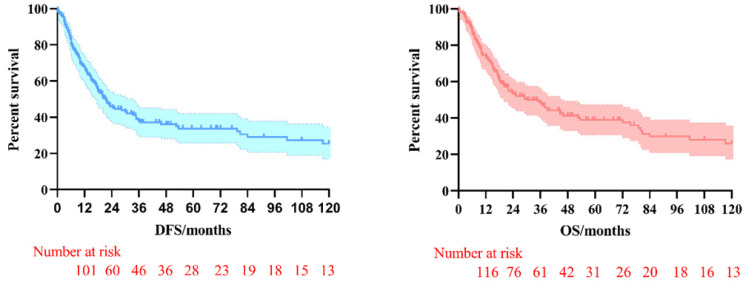
The survival curves of DFS and OS in pT4b gastric cancer patients who underwent MVR surgery.

**Figure 2:**
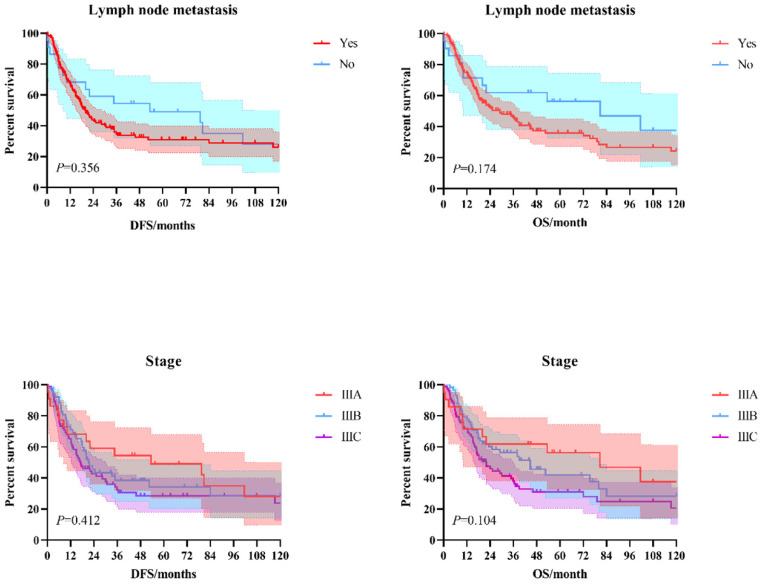
The survival curves of DFS and OS according to the lymph node metastasis status and TNM stage.

**Figure 3:**
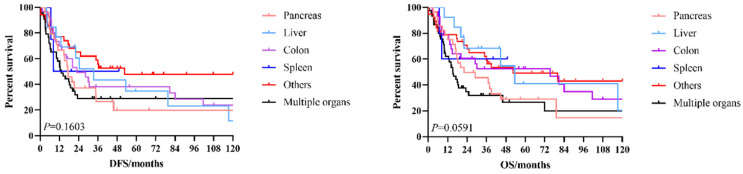
The survival curves of DFS and OS according to the combined resection organs.

The 3-year and 5-year disease-free survival (DFS) rates for the patients investigated were 38.0% and 33.8%, respectively, and the 3-year and 5-year overall survival (OS) rates were 48.2% and 39.1%, respectively. The survival curves of DFS and OS were shown in **Figure 1**. Subgroup survival curve analysis according to the lymph node metastasis status and TNM stage found no statistical differences (**Figure 2**). Furthermore, we compared the survival curves of DFS and OS according to the combined resection organs and found that the patients received combined resection of pancreas and multiple organs were tend to have worse survival (**Figure 3**).

When conducting the multivariate Cox regression survival analysis, we found that negative nerve invasion was independent risk factors for DFS (HR: 2.202, 95%CI: 1.144-4.236, *P*=0.018) (**Table 3**) and OS (HR: 2.219, 95%CI: 1.164-4.231, *P*=0.015) (**Table 4**).

### Recurrence pattern

A total of 114 patients (54.3%) developed recurrences. Additionally, 28 patients were followed up for over five years and no evidence of recurrence were found. Among the 34 patients with documented recurrence, 18 patients developed only locoregional recurrence, 2 patients developed distant metastasis, and 8 patients developed only peritoneal metastasis. The specific recurrence sites were demonstrated in **Table 5**.

## Discussions

Radical surgery (R0) is the only potentially curable treatment for GC patients. For pT4b GC patients, MVR surgery is necessary to achieve R0 resection. However, this operation remains debatable due to surgical difficulty and high incidence of postoperative complications and mortality. Therefore, we integrated 20-year experience to explore this clinically important question. Our results demonstrated that MVR surgery had relatively acceptable short-term safety and long-term efficacy. The current study provides further evidence to support MVR surgery in the future clinical practice to some extent.

The rates of postoperative complications and mortality were 8.1% and 0.5% in the present study, which were relatively lower than the previous studies. Previous studies indicated that the postoperative complications rate ranged from 15% to 53.4%, and mortality rate ranged from 1.0% to 8.6% 2, 4, 6, 7, 9-11, 15, 16. Such discrepancy might have several reasons. Firstly, regional difference in GC prevalence might contribute to the different risk of surgical complications and death. In areas of high GC incidence, such as Korea, the postoperative complications and mortality rates of MVR surgery were 15% and 2% 11. However, in Brazil, the postoperative complications and mortality rates of MVR surgery could achieve 53.4% and 8.6% 7. Secondly, the difference of combined resection organs might lead to the different results. Several investigators have suggested that combined pancreas resection in MVR surgery of T4b GC patients could arise the risk of postoperative complications and mortality 11. Although Tran et al. found that MVR surgery with pancreas resection was not associated with an increased frequency of postoperative complications and mortality 10. The gastrectomy combined with pancreaticoduodenectomy was considered to have high risk of postoperative death and complications 3, 10. Thirdly, the number of cases in the present study and previous studies were relatively small, which could potentially bias the results.

Several studies have showed a substantial survival advantage of MVR surgery in T4b GC patients, comparing with gastrectomy alone or palliative surgery 6, 17-20. However, some other studies have demonstrated that GC patients with MVR surgery achieved worse survival than those with gastrectomy alone 7, 15. Overall, the 5-year OS rate of MVR surgery for GC patients was 13.8%~36.8% 2, 4, 11, 15, 17, 20. In the present study, the 5-year OS rate of pT4b GC patients who received MVR surgery was 39.1%, which was a little higher than the previous results. This may be related to the fact that the previous results have illustrated that prognosis in the patients combined with pancreas resection, especially in patients who underwent pancreaticoduodenectomy, was poor than patients combined other organs resection during MVR surgery 3, 10, 11. In a Korea retrospective study, the 5-year OS rates of pT4b patients combined pancreas resection group and combined other organs resection were 23.3% and 42.1% (*P*=0.002), while in the pancreaticoduodenectomy group, the 5-year OS rates was 0% 11. Similarly, a significant survival difference among pT4b patients without and with pancreas resection was reported in Taiwan (32% vs. 13%, *P*=0.004). However, in the current study, approximately one in five patients underwent combined pancreas resection alone and only four patients underwent gastrectomy combined pancreaticoduodenectomy surgery.

It was worth noting that in our study, we found that the pN stage was not the independent prognostic factor in GC patients who received MVR surgery. This is in contrast with previous findings 2, 4, 6, 10, 11, 15, 21. A possible reason for such contrary results may be that the current pN stage has insufficient homogeneity and discriminatory ability in predicting the survival of GC patients comparing with the positive lymph nodes ratio 22.

Previous studies have shown that the majority recurrence occurred within two years after surgery in GC patients 23, 24. Moreover, Zhu BY et al. found that 43.8% GC patients with T4 stage experienced recurrence after curative surgery, and peritoneal metastasis was the major recurrence pattern accounting for 62.2% 8. Furthermore, for pT4b patients, the proportion of peritoneal metastasis even reached to 88.3% 8. However, in the present study, 54.3% pT4b GC patients after MVR surgery developed recurrence, and the major recurrence pattern were locoregional recurrence. Possibly, this difference in results could be related to the fact that the first sites of recurrence were not recorded in many patients in our study.

Indeed, we do acknowledge that there were some limitations in this study. Firstly, some key data and clinical information were missing for some patients due to the retrospective nature of our study. Secondly, follow-up time was relatively short in some patients, and some patients were lost to follow-up. Thirdly, many patients were followed by other oncological centers, therefore, the adjuvant treatment strategies and first sites of recurrence were not well recorded. Despite this, we believe that our study has unique features and certain strengths. Firstly, our study mainly targeted pT4b GC patients, which avoided the interference of some pT4a GC patients. Secondly, to the best of our knowledge, the current study was the largest sample size study in China that have assessed the short-term safety and long-term efficacy of potential curative MVR surgery in pT4b GC patients.

## Conclusions

In our study, we found that the rates of postoperative complications and mortality were 8.1% and 0.5%, and the 3-year and 5-year overall survival rates were 48.2% and 39.1% in pT4b gastric cancer patients without distant metastasis after MVR surgery. Therefore, MVR surgery in pT4b gastric cancer patients without distant metastasis was effective and had an acceptable safety profile.

## Figures and Tables

**Table 1:** Clinicopathologic features of pT4b gastric cancer patients who underwent MVR surgery

Characteristic	n=210	100%
**Age (mean±SD)**	61±11	
≤65	138	65.7%
>65	72	34.3%
**Gender**		
Male	153	72.9%
Female	57	27.1%
**Tumor location**		
Proximal	110	52.4%
Distal	87	41.4%
Total	13	6.2%
**Neoadjuvant therapy**		
No	189	90.0%
Yes	21	10.0%
**Gastric stump carcinoma**		
No	145	69.0%
Yes	24	11.4%
Unknown	41	19.5%
**Surgical approach**		
Open	196	93.3%
Laproscope	14	6.7%
**Gastric surgery**		
Total gastrectomy	30	14.3%
Sub gastrectomy	180	85.7%
**Tumor size (pathology)**		
<5cm	62	29.5%
≥5cm	148	70.5%
**Differentiation**		
Well and Moderate	49	23.3%
Poor and Undifferentiated	161	76.7%
**Borrman classification**		
I	14	6.7%
II	45	21.4%
III	98	46.7%
IV	46	21.9%
Unknown	7	3.3%
**Lauren classification**		
Intestinal type	28	13.3%
Diffuse type	30	14.3%
Mixed type	23	11.0%
Unknown	129	61.4%
**pN stage**		
N0	27	12.9%
N1	26	12.4%
N2	53	25.2%
N3	104	49.5%
**pTNM stage**		
IIIA	27	12.9%
IIIB	79	37.6%
IIIC	104	49.5%
**Lymphatic vessels invasion**		
Positive	104	49.5%
Negative	106	50.5%
**Blood vessels invasion**		
Positive	105	50.0%
Negative	105	50.0%
**Nerve invasion**		
Positive	65	31.0%
Negative	145	69.0%
**Margin involved**		
R0	198	94.3%
R1/R2	12	5.7%
**Combined organs removed**		
Pancreas	43	20.5%
Liver	19	9.0%
Colon	35	16.7%
Spleen	5	2.4%
Other (abdominal wall, diaphragm, gallbladder, kidney)	44	21.0%
Multiple organs	64	30.5%
**Blood transfusion**		
Yes	99	47.1%
No	111	52.9%
**Postoperative complications**		
No	193	91.9%
Yes	17	8.1%
**Adjuvant treatment**		
Yes	81	38.6%
No	15	7.1%
Unknown	114	54.3%

**Table 2:** Operative difficulty and short-term safety of pT4b gastric cancer patients who underwent MVR surgery

Variables	Total (n=210)	Spleen (n=5)	Colon (n=35)	Pancreas (n=43)	Liver (n=19)	Other^*^ (n=44)	Multiple organs (n=64)	*P*-value
Mean operative time /min	209.8	250.8	199.6	209	209.6	184.8	222.3	0.057
**Blood transfusion**								<0.001
No	99	3	20	14	9	10	42	
Yes	111	2	15	27	20	34	22	
Blood transfusion /ml	890.8	1500	878	871.9	968.4	740	927.5	0.201
Mean postoperative hospital stay /d	16.5	18.4	16.8	19	17.1	11.9	18.5	<0.001
**Postoperative complications**								0.007
No	193	3	33	39	19	44	55	
Yes	17	2	2	4	0	0	9	
**Anastomotic leak**	9	1	2	2	0	0	4	
**Intra-abdominal infections**	12	2	2	2	0	0	6	
**Intra-abdominal hemorrhage**	1	0	0	0	0	0	1	
**Gastrointestinal hemorrhage**	1	0	0	0	0	0	1	
**Postoperative intestinal obstruction**	0	0	0	0	0	0	0	
**Gastroparesis**	0	0	0	0	0	0	0	
**Pulmonary complications**	4	0	0	2	0	0	2	
**Pancreatic fistula**	1	0	0	1	0	0	0	

*Including: abdominal wall, diaphragm, gallbladder, kidney.

**Table 3:** Univariate and multivariate Cox regression analysis for disease-free survival of pT4b gastric cancer patients who underwent MVR surgery

Characteristic	Univariate analysis		Multivariate analysis	
	HR [95%CI]	*P-value*	HR [95%CI]	*P-value*
**Age**				
≤65	Reference			
>65	0.809[0.519-1.262]	0.350		
**Gender**				
Male	Reference			
Female	1.266[0.803-1.996]	0.310		
**Tumor location**				
Proximal	Reference			
Distal	1.006[0.669-1.512]	0.978		
Total	0.627[0.250-1.574]	0.320		
**Neoadjuvant therapy**				
No	Reference			
Yes	0.983[0.494-1.958]	0.962		
**Gastric stump carcinoma**				
No	Reference			
Yes	0.706[0.323-1.541]	0.382		
Unknown	1.067[0.672-1.694]	0.784		
**Surgical approach**				
Open	Reference			
Laproscope	1.305[0.630-2.702]	0.473		
**Gastric surgery**				
Total gastrectomy	Reference			
Sub gastrectomy	0.976[0.543-1.756]	0.936		
**Tumor size (pathology)**				
<5 cm	Reference		Reference	
≥5 cm	1.586[1.022-2.461]	0.040	1.372[0.862-2.184]	0.183
**Differentiation**				
Well and Moderate	Reference			
Poor and Undifferentiated	0.928[0.571-1.507]	0.762		
**Borrman classification**				
I	Reference			
II	1.401[0.488-4.024]	0.531		
III	1.066[0.381-2.980]	0.903		
IV	1.621[0.561-4.686]	0.372		
Unknown	1.436[0.261-7.883]	0.677		
**Lauren classification**				
Intestinal type	Reference		Reference	
Diffuse type	0.860[0.434-1.704]	0.665	0.738[0.336-1.622]	0.450
Mixed type	0.504[0.199-1.280]	0.150	0.408[0.143-1.163]	0.093
Unknown	1.043[0.604-1.802]	0.879	0.615[0.295-1.284]	0.196
**pN stage**				
N0	Reference		Reference	
N1	2.271[1.067-4.835]	0.033	1.471[0.624-3.468]	0.378
N2	0.862[0.431-1.723]	0.675	0.748[0.343-1.629]	0.464
N3	1.442[0.795-2.617]	0.228	1.311[0.665-2.586]	0.434
**pTNM stage**				
IIIA	Reference			
IIIB	1.157[0.617-2.170]	0.649		
IIIC	1.426[0.786-2.586]	0.243		
**Lymphatic vessels invasion**				
Positive	Reference		Reference	
Negative	0.743[0.498-1.108]	0.145	2.353[0.278-19.942]	0.433
**Blood vessels invasion**				
Positive	Reference		Reference	
Negative	0.728[0.488-1.086]	0.120	0.295[0.034-2.572]	0.269
**Nerve invasion**				
Positive	Reference		Reference	
Negative	1.666[1.071-2.590]	0.024	2.202[1.144-4.236]	0.018
**Margin involved**				
R0	Reference			
R1/R2	1.492[0.721-3.088]	0.281		
**Combined organs removed**				
Pancreas	Reference		Reference	
Liver	0.791[0.364-1.719]	0.554	0.842[0.370-1.916]	0.681
Colon	0.764[0.406-1.441]	0.406	0.700[0.361-1.358]	0.292
Spleen	0.752[0.174-3.245]	0.703	1.233[0.242-6.278]	0.801
Other (abdominal wall, diaphragm, gallbladder, kidney)	0.494[0.254-0.962]	0.038	0.710[0.334-1.509]	0.373
Multiple organs	1.104[0.615-1.981]	0.741	0.985[0.529-1.834]	0.963
**Blood transfusion**				
Yes	Reference			
No	0.974[0.655-1.448]	0.897		
**Postoperative complications**				
No	Reference		Reference	
Yes	1.803[0.937-3.471]	0.078	1.461[0.709-3.010]	0.304
**Adjuvant treatment**				
Yes	Reference		Reference	
No	0.806[0.344-1.888]	0.619	0.536[0.209-1.377]	0.195
Unknown	1.015[0.673-1.529]	0.944	1.026[0.649-1.623]	0.911

**Table 4:** Univariate and multivariate Cox regression analysis for overall survival of pT4b gastric cancer patients who underwent MVR surgery

Characteristic	Univariate analysis		Multivariate analysis	
	HR [95%CI]	*P-value*	HR [95%CI]	*P-value*
**Age**				
≤65	Reference			
>65	0.923[0.597-1.426]	0.717		
**Gender**				
Male	Reference			
Female	1.260[0.798-1.991]	0.321		
**Tumor location**				
Proximal	Reference			
Distal	0.965[0.643-1.449]	0.864		
Total	0.626[0.249-1.569]	0.317		
**Neoadjuvant therapy**				
No	Reference			
Yes	0.839[0.446-1.580]	0.587		
**Gastric stump carcinoma**				
No	Reference			
Yes	0.985[0.539-1.800]	0.961		
Unknown	1.033[0.646-1.651]	0.893		
**Surgical approach**				
Open	Reference			
Laproscope	1.046[0.505-2.167]	0.903		
**Gastric surgery**				
Total gastrectomy	Reference			
Sub gastrectomy	0.813[0.459-1.443]	0.480		
**Tumor size (pathology)**				
<5cm	Reference		Reference	
≥5cm	1.613[1.043-2.495]	0.032	1.410[0.882-2.254]	0.151
**Differentiation**				
Well and Moderate	Reference		Reference	
Poor and Undifferentiated	0.742[0.476-1.156]	0.188	0.619[0.361-1.062]	0.082
**Borrman classification**				
I	Reference		Reference	
II	1.310[0.547-3.139]	0.545	1.222[0.459-3.252]	0.688
III	1.103[0.488-2.498]	0.813	1.224[0.470-3.187]	0.679
IV	1.852[0.776-4.421]	0.165	1.623[0.584-4.514]	0.353
Unknown	2.076[0.646-6.672]	0.220	2.695[0.718-10.107]	0.142
**Lauren classification**				
Intestinal type	Reference		Reference	
Diffuse type	1.098[0.516-2.337]	0.808	1.041[0.433-2.502]	0.928
Mixed type	0.942[0.402-2.205]	0.890	0.766[0.290-2.024]	0.591
Unknown	1.642[0.896-3.010]	0.109	0.871[0.386-1.969]	0.741
**pN stage**				
N0	Reference		Reference	
N1	1.697[0.722-3.988]	0.225	1.218[0.483-3.071]	0.677
N2	1.108[0.548-2.242]	0.776	0.760[0.342-1.691]	0.502
N3	1.750[0.930-3.293]	0.083	1.646[0.803-3.371]	0.173
**pTNM stage**				
IIIA	Reference			
IIIB	1.234[0.632-2.409]	0.539		
IIIC	1.743[0.927-3.279]	0.085		
**Lymphatic vessels invasion**				
Positive	Reference			
Negative	0.777[0.520-1.162]	0.219		
**Blood vessels invasion**				
Positive	Reference			
Negative	0.809[0.541-1.209]	0.301		
**Nerve invasion**				
Positive	Reference		Reference	
Negative	2.131[1.330-3.413]	0.002	2.219[1.164-4.231]	0.015
**Margin involved**				
R0	Reference			
R1/R2	1.244[0.574-2.696]	0.580		
**Combined organs removed**				
Pancreas	Reference		Reference	
Liver	0.573[0.242-1.356]	0.205	0.814[0.313-2.113]	0.672
Colon	0.683[0.357-1.308]	0.251	0.711[0.354-1.428]	0.337
Spleen	0.641[0.150-2.742]	0.548	0.998[0.196-5.075]	0.998
Other (abdominal wall, diaphragm, gallbladder, kidney)	0.574[0.307-1.076]	0.083	0.923[0.448-1.899]	0.828
Multiple organs	1.265[0.722-2.215]	0.412	1.270[0.691-2.336]	0.442
**Blood transfusion**				
Yes	Reference			
No	0.939[0.629-1.400]	0.756		
**Postoperative complications**				
No	Reference		Reference	
Yes	1.606[0.834-3.093]	0.156	1.704[0.761-3.816]	0.195
**Adjuvant treatment**				
Yes	Reference		Reference	
No	1.140[0.523-2.482]	0.742	0.830[0.342-2.014]	0.681
Unknown	1.488[0.981-2.257]	0.061	1.792[1.113-2.885]	0.016

**Table 5:** The specific recurrence sites of pT4b gastric cancer patients who underwent MVR surgery

Recurrence and Metastasis sites	N=114	54.3%
**Specific sites that were well recorded**	34	16.2%
Liver metastasis	4	1.9%
Peritoneal metastasis	9	4.3%
Locoregional areas recurrence	11	5.2%
Supraclavicular lymph nodes metastasis	1	0.5%
Remnant stomach recurrence	12	5.7%
Ovarian metastasis	2	1.0%
Lung metastasis	2	1.0%
Brain metastasis	1	0.5%
**Specific sites that were not recorded**	80	38.1%
